# Macrophage Polarization in Leishmaniasis: Broadening Horizons

**DOI:** 10.3389/fimmu.2018.02529

**Published:** 2018-10-31

**Authors:** Fernanda Tomiotto-Pellissier, Bruna Taciane da Silva Bortoleti, João Paulo Assolini, Manoela Daiele Gonçalves, Amanda Cristina Machado Carloto, Milena Menegazzo Miranda-Sapla, Ivete Conchon-Costa, Juliano Bordignon, Wander Rogério Pavanelli

**Affiliations:** ^1^Biosciences and Biotechnology Postgraduate Program, Carlos Chagas Institute (ICC), Fiocruz, Curitiba, Brazil; ^2^Laboratory of Immunoparasitology, Department of Pathological Sciences, State University of Londrina, Londrina, Brazil; ^3^Laboratory of Biotransformation and Phytochemistry, Department of Chemistry, State University of Londrina, Universitary Hospital, Londrina, Brazil; ^4^Laboratory of Molecular Virology, Carlos Chagas Institute (ICC), Fiocruz, Curitiba, Brazil

**Keywords:** classical macrophage, non-classical macrophage, vector saliva, *Leishmania*, immunomodulation, chemokine

## Abstract

Leishmaniasis is a vector-borne neglected tropical disease that affects more than 700,000 people annually. *Leishmania* parasites cause the disease, and different species trigger a distinct immune response and clinical manifestations. Macrophages are the final host cells for the proliferation of *Leishmania* parasites, and these cells are the key to a controlled or exacerbated response that culminates in clinical manifestations. M1 and M2 are the two main macrophage phenotypes. M1 is a pro-inflammatory subtype with microbicidal properties, and M2, or alternatively activated, is an anti-inflammatory/regulatory subtype that is related to inflammation resolution and tissue repair. The present review elucidates the roles of M1 and M2 polarization in leishmaniasis and highlights the role of the salivary components of the vector and the action of the parasite in the macrophage plasticity.

## Introduction

Leishmaniasis is a broad term that is used for a group of vector-borne diseases caused by species of protozoan parasites of the *Leishmania* genus of which 18 spp. are pathogenic to humans (1). The disease presents in five main clinical forms: visceral leishmaniasis (VL, or kala-azar), cutaneous leishmaniasis (CL), mucocutaneous leishmaniasis (MCL), diffuse cutaneous leishmaniasis (DCL) and post-kala-azar dermal leishmaniasis (PKDL) (2). All types of leishmaniases are transmitted to an animal or human reservoir through the bite of female infected phlebotomine sand flies, which infect a range of 70 animal species, including humans, rodents, and canids in their transmission cycle (3).

The World Health Organization (WHO) classifies leishmaniasis as a neglected tropical disease because it is directly linked to economically disadvantaged populations in tropical regions (2). A total of 700,000 to one million cases of leishmaniasis occur annually in 102 countries, areas or territories worldwide, with 20,000–30,000 deaths (3).

The high prevalence of this disease is directly influenced by the success of long host–parasite coevolutionary process in which parasites *Leishmania* have the ability to manipulate the vertebrate immune system in their favor, through the synthesis of parasites molecules, but also by vector saliva molecules, which are injected into the blood-feeding site during transmission (4).

The *Leishmania* parasites exhibit a biological digenetic life cycle with variable morphology that alternates between two main distinct developmental stages: the free-living flagellated promastigote form found in the midgut of phlebotomine sandfly vectors and the obligate intracellular aflagellated amastigotes in phagolysosomal vesicles of the vertebrate phagocytic cells, mainly into macrophages (5, 6).

During the blood feeding of the infected sandfly, which inoculates the host with metacyclic promastigotes and a large portion of the salivary content of the insect. Phlebotomine saliva is composed of pharmacologically active components with anti-hemostatic, chemotactic and immunomodulatory properties, that directly influence the parasite infection process modulating the local immune response (7). At the site of the bite occurs a rapid and intense neutrophil infiltration after inoculation, followed by monocytes/macrophages (8, 9).

Neutrophils primarily phagocytize most (80–90%) of the parasites and produce chemokines and cytokines that recruit and activate different cell types to regulate the development of the adaptive immune response during *Leishmania* sp. infection (8). Neutrophils are important components of the initial immune response against *Leishmania* parasites, even though there are currently contradictory findings on their role in the *Leishmania* infection.

Although the effective participation of neutrophils in the elimination of the parasite has been reported for *L. braziliensis, L. amazonensis* (10–15) and *Leishmania donovani* (16), collectively, most of these studies reported that the leishmanicidal action of neutrophils is clearly insufficient to control the establishment of infection and the development of the disease [reviewed in (17)].

Subversion of neutrophil killing functions by *Leishmania* is a strategy that allows parasite spreading in the host with a consequent infection evolution, transforming the primary protective role of neutrophils into a deleterious one. Neutrophils do not eliminate the parasite but act as “Trojan horses,” becoming late apoptotic and rapidly internalized by macrophages and dendritic cells, increasing the infectivity and persistence of the parasite (18, 19).

Macrophages play a dual role in *Leishmania* infection. These cells are responsible for the destruction of internalized parasites but also provide a safe place for *Leishmania* replication. Therefore, macrophages are key to disease progression and the success or failure of the infection depends on the interplay between infecting *Leishmania* species and the type and magnitude of the host's immune response. Both of these factors are closely related to the clinical forms of leishmaniasis (20, 21).

Macrophages are normally at rest as naïve macrophages (M0), but the microenvironment in which these cells are found provides different signals that activate them and lead to the development of functionally distinct macrophage's phenotype, toward “classically activated” (M1) or “alternatively activated” (M2) with different disease outcomes (22, 23). Therefore, the activation of M1 macrophages by Th1 lymphocyte subpopulation, which produces various cytokines, primarily interferon gamma (IFN-γ) and tumor necrosis factor-alpha (TNF-α) is crucial for the elimination of this intracellular pathogen via the triggering of an oxidative burst. The host cells increase the production of reactive oxygen species (ROS), including superoxide, hydrogen peroxide, and hydroxyl radicals, and nitric oxide (NO), which exhibit high microbicidal capacity (20, 22).

In contrast, the activation of Th2 lymphocytes, which produce IL-4 and IL-13 cytokines, induces the M2 profile caractherized by polyamine biosynthesis via activation of the enzyme arginase (arg) and production of urea and *L*-ornithine, which are beneficial for *Leishmania* intramacrophage growth favoring parasite survival in the infected macrophages and disease progression (22, 24).

Different *Leishmania* species trigger distinct immune responses (25). These responses are far beyond the classical Th1/Th2 paradigm (26) and increase the interest in understanding the role of M1 and M2 macrophages in the context of different *Leishmania* species infection. The immunomodulatory influence of the saliva of different leishmaniasis vectors should also be considered in the differential recruitment/activation of macrophages subtypes.

It is known how important macrophages are in the resistance or susceptibility to *Leishmania* infection, therefore we reviewed the impact of macrophage plasticity and M1 and M2 phenotypes on infection outcome. We also consider the role of vector saliva, which is a well-established immunomodulatory element in the *Leishmania* infection, in macrophage plasticity and phenotype.

## M1 and M2 macrophages: an overview

Macrophages are phagocytic cells that are found in several tissues. In innate immunity, macrophages are responsible primarily for the control of pathogens and in adaptive immunity, this cell participates in the recognition, processing, and presentation of antigens to T cells (27). Macrophages interact with T and B cells via intercellular contact and the production of molecules and mediators to participate in the inflammatory response and tissue repair (27).

Two main macrophages phenotypes are known, M1 and M2 (28). The M1, or the classically activated macrophage, is a pro-inflammatory subtype that exhibits microbicidal properties (29). The M2, or the alternatively activated macrophage, is an anti-inflammatory/regulatory subtype that plays a role in the resolution of inflammation and tissue repair (30). The polarization of macrophages into M1 and M2 phenotypes is dependent on the signals provided by the microenvironment (28, 30, 31).

The designations M1 and M2 originated from Th1 and Th2 cytokine patterns and are associated with the change in macrophage phenotypes (32). Macrophage subtypes also differ in the production of cytokines, chemokines and other mediators and the expression of receptors, surface molecules, and transcription factors, which can act as specific markers to aid in the identification and function of these cells (27, 29, 32–34).

M1 macrophages are characterized by a high production of pro-inflammatory cytokines (TNF-α, IL-1β, IL-6, IL-12, IL-18, IL-23, and Type 1 IFN), high phagocytosis rate, and the production of reactive oxygen and nitrogen species and act to control intracellular pathogens. This macrophage subtype plays a role in tumor control, and it may be involved in autoimmune diseases and tissue damage (7, 27, 34–39).

M1 macrophages constitute the first line of defense against intracellular pathogens and induce the development of the Th1 response via IL-12 secretion (40, 41). The polarization of M1 macrophages may be primarily due to the presence of lipopolysaccharide (LPS), IFN-γ or TNF-α. Granulocyte-macrophage colony-stimulating factor (GM-CSF) may also result in the differentiation and maintenance of the M1 phenotype (41, 42). Ruan et al. (43) demonstrated that complement system activation is also related to M1 polarization.

M1 polarization activates transcription factors, such as AP1, STATs, NFκBp65 and IRFs, which lead to the expression of pro-inflammatory genes, costimulatory molecules and chemokines to attract various immune cells (Table 1) (7, 27, 34–39). IRF4 and IRF5 are involved in the polarization of M1 and M2 macrophages, but the role of IRFs in M2 polarization is not completely clear (44).

**Table 1:** Chemokines differentially produced by M1 and M2 macrophages and their role in cell recruitment.

**M1 macrophages**		**M2 macrophages**	
**Chemokines**	**Cell recruitment**	**Chemokines**	**Cell recruitment**
CXCL1	Neutrophils	CXCL13	B cells
CXCL2	Granulocytes, polymorphonuclear	CCL1	Monocytes, Th2 and Treg cells
CXCL3	Neutrophils	CCL16	Monocytes and lymphocytes
CXCL5	Neutrophils	CCL17	Th2 cells
CXCL8	Neutrophils	CCL18	Th2 cells
CXCL9	Activated T cells	CCL22	Th2 and Treg cells
CCL2	Monocytes, memory T cells and NK	CCL24	Eosinophils and basophils
CCL3	Monocytes, T lymphocytes and polymorphonuclear cells	
CCL4	Monocytes, T lymphocytes and polymorphonuclear cells	
CCL11	Eosinophils	
CX3CL1	T cells and monocytes	

*CCL, CC-chemokine ligand; CXCL, CXC-chemokine ligand; NK, natural killer cells*.

The alternatively activated macrophages, or M2, exhibit an anti-inflammatory/immunoregulatory phenotype that is related to tissue remodeling and repair, resistance to some parasites and the promotion of tumor growth (30, 45, 46). M2 was initially characterized by the expression of mannose receptor (CD206), but a range of markers and mediators produced by these cells were described, including important chemokines for the recruitment of different cells (Table 1) (37, 45, 47).

Different stimuli, such as IL-4/IL-13, IL-10, TGF-β, M-CSF, vitamin D3, and immunocomplexes, induce M2 macrophages (48, 49). Other cytokines, such as IL-21 and IL-33, may also act on macrophage polarization and maintenance to an M2 phenotype (50–52). Li et al. (52) demonstrated that IL-21 reduced the expression of CD86, iNOS, TLR-4, and IL-6 and TNF-α production via STAT3 phosphorylation. IL-33 amplifies IL-13-induced M2 polarization (50, 51).

The classification of M2 macrophages was recently expanded and subdivides these cells into four subtypes, M2a, M2b, M2c, and M2d, according to stimulus and function (23, 45). M2a macrophages are polarized by macrophage colony-stimulating factor (M-CSF) and IL-4 or IL-13. This subtype is characterized by arg-1, IL-10, and SOCS3 expression and produces CCL24, CCL17, and CCL22, which are responsible for the recruitment of eosinophils, basophils and Th2 cells. These cells are involved in allergic reactions, parasite death and encapsulation, the promotion of fibrogenesis, tissue repair and cell proliferation (53–55).

The M2b phenotype is induced by the combination of immunocomplexes with IL-1βRa/TLR ligands, apoptotic cells or LPS. These cells secrete a large amount of IL-10 and inflammatory mediators, such as TNF-α and IL-6, and express iNOS (30, 53). M2b macrophages secrete the CCL1 chemokine, which results in the infiltration of eosinophils, Th2 lymphocytes and regulatory T cells (Tregs) (56). Therefore, M2b macrophages act as an immunoregulator and trigger activation of the Th2 response (23, 53).

M2c macrophage polarization results from IL-10, TGF-β and glucocorticoids (38, 57, 58). This subtype produces IL-10, TGF-β, CXCL13, CCL16, and CCL18, which leads to the down-regulation of pro-inflammatory cytokines and an increase in the recruitment of eosinophils and naïve T cells. M2c cells express high levels of arg-1, CD163, CD206, scavenger receptors, TLR1, TLR8, FPR1, CCR2, and CCR5, and this subtype is involved in tissue regeneration and angiogenesis (53–55, 59).

The M2d macrophage phenotype is involved in the inhibition of the immune response and the promotion of angiogenesis (60). These cells are induced by IL-6, toll-like receptor (TLR) ligands and adenosine A_2A_ receptor agonists (60, 61). Stimulation with adenosine may result in the polarization of M1 macrophages to M2d (53, 61). This phenotype expresses high levels of IL-10, VEGF, and iNOS and secretes CCL5, CXCL10, and CXCL6 and low levels of IL-12 and TNF-α (53, 60–63).

Signals of the microenvironment are of great importance of the change in the polarization state of macrophages, and the programming from one phenotype to another is closely related to the activation of specific transcription factors and microRNAs (miRNAs) (64, 65). miRNAs are small molecules of non-coding RNAs which can act on gene expression at the post-transcriptional level (64), regulating some important transcription factors in the M1 and M2 phenotypes (66, 67).

Li et al. (67) reviewed the role of various miRNAs which participate in the regulation and polarization of macrophages in murine and human models. The miRNAs miRNA-9, miRNA-146a, miRNA-146b, miRNA-124, miRNA-181a, miRNA let-7c, miRNA-93, and miRNA-210 act to suppress the M1 and promote M2 phenotype. On the other hand, miRNA-27a, miRNA-130a, miRNA-130b, miRNA-155, miRNA-21, miRNA-19a-3p, miRNA-23a, miRNA-125a, miRNA-125b, miRNA-26a, miRNA-26b, and miRNA-720 act on transcription factors involved in the promotion of M1 phenotype and M2 suppression [reviewed in (67)].

Although we discussed above on the macrophage polarization of M0 to M1 or M2, it is known that macrophages have high plasticity and can be repolarized or reprogrammed under specific stimuli, in other words, M1 macrophages can differentiate into M2, and vice versa (40, 68). M2 macrophage may be repolarized more quickly than M1 macrophages, and this repolarization can occur through exposure of TLR ligands such as LPS and/or IFN-γ, or by expression of miRNAs such as miR-155 (69, 70). Van den Bossche et al. (71) showed that exposure of M1 macrophages to IL-4 is not able to reprogram the macrophages to M2 (71). However, the miRNAs can suppress the M1 phenotype and promote the polarization to M2 as commented above (67).

## Interaction of vector saliva with immune cells

Among over 800 species of phlebotomines recorded, 98 are proven or suspected vectors of human leishmaniases; these include 42 *Phlebotomus* species in the Old World and 56 *Lutzomyia* species in the New World (all: Diptera: Psychodidae) (72). It is known that through the insect bite, vector saliva plays an important role in the establishment of *Leishmania* infection by increasing the infectivity of the parasite and modulating the host immune response (73, 74). Arthropod saliva contains anti-inflammatory, chemotactic and anti-hemostatic components that influence the course of parasite transmission to the host (7, 75, 76).

Most studies of the role of vector saliva in disease course were performed prior to the establishment of the M1 and M2 macrophage concept. Therefore, these works do not use this nomenclature. However, they investigated molecules that are involved in the plastic response of macrophages. These molecules are discussed below.

Some groups produced extracts, homogenates, sonicates and salivary lysates to elucidate the function of vector saliva in *Leishmania* transmission and infection (Table 2). We review the literature and assemble the results of different groups to provide an overview of the function of saliva.

**Table 2:** Salivary compounds and their effects on *Leishmania* infection.

**Compound**	**Immunomodulatory effect**		**References**
Promastigote secretory gel (PSG)	↑ Arg ↑ IL-1β ↑ IL-6 ↑ IL-10 ↑ TNF-α ↑ CCL2 ↑ CCL4 ↑ CCL3 ↑ CXCL2 ↑ FGFR2 ↑ EGF ↑ EGFR ↑ IGF1		(77) 78)
Salivary Gland Homogenate (SGH)	↑ MCP-1 ↑ CCR2 ↑ IL-10 ↑ Eosinophils ↑ Macrophages ↑ IFN-γ ↑ IL-13 ↑ IL-5	↓ iNOS ↓ NO ↓ IFN-γ ↓ IL-13 ↓ IL-5	(79) (80, 81) (82)
Salivary Gland Lysate (SGL)	↑ IL-4 ↑ IL-6	↓ IFN-γ ↓ IL-12 ↓ iNOS	(83, 84) (85–88)
Salivary Gland Extracts (SGE)	↑ IL-10 ↑ IL-4 ↑ CD8 ↑ INF-γ ↑ CD4	↓ NO	(89) (90)
Salivary Gland Sonicate (SGS)	↑ IL-4 ↑ PGE_2_ ↑ Macrophages ↑ LTB_4_	↓ IFN-γ	(91, 92)
Maxadilan (max)	↑ IL-6 ↑ IL-10 ↑ TGF-β ↑ CD86	↓ IL-1β ↓ IL-12p70 ↓ TNF-α ↓ IFN-γ ↓ CD80 ↓ CCR7	(87, 93, 94)
Adenosine	↑ IL-10		(73)
	↑ PGE_2_		

*CCR, chemokine receptor; CD, cluster of differentiation; IFN, interferon; IL, interleukin; iNOS, inducible nitric oxide synthase; MCP-1, monocyte chemoattractant protein-1; NO, nitric oxide; PG, prostaglandin; TNF, tumor necrosis factor; TGF, transforming growth factor; CCL, chemokines; CXCL, motif chemokine ligand; FGFR, fibroblast growth factor receptor; EGF, epidermal growth factor; EGFR, epidermal growth factor; IGF, Insulin-like growth factor*.

Saliva contains molecules that induce a long-lasting erythema, which facilitates the obtaining of blood from capillaries in the host tissue (79). Vector saliva may facilitate cell recruitment via the promotion of vasodilation (79, 80, 92). The recruited cells include neutrophils, eosinophils and macrophages. Neutrophils may be attracted by the presence of several mediators, such as LTB_4_, and the role of these cells as a “Trojan horse” favors the establishment of infection (80, 92). The role of eosinophils is controversial, and whether these cells promote or suppress the infection is not well known (80, 95).

Some studies observed that vector saliva increased macrophages recruitment to the site of infection because of the modulation of chemotactic factors, such as CCL2/MCP-1, CCR2 and PGE_2_ (80, 92). Although some studies have shown the participation of lipid mediators such as PGE_2_ and LTB_4_ in cell migration (73, 92), the role of these lipid mediators acting in the polarization or recruitment of a specific profile of macrophages is uncertain, differing between cell types and models studied (28, 96–98).

Vector saliva plays an important immunomodulatory role and favors the M2 profile in different manners. Vector saliva induces IL-10 to promote a regulatory response (81, 89, 90), and it is related to the activation of a Th2 response via the increase in IL-4 and IL-6 synthesis (85, 87) Rohousov et al. (88). Besides that, the salivary components reducing the M1 related parameters, such as the pro-inflammatory cytokines IFN-γ and IL-12, iNOS and nitric oxide (NO) (85, 86, 88, 90).

However, some studies showed the action of saliva inducing M1 parameters. C57BL/6 mice immunized with plasmids encoding salivary proteins developed a Th1 response, resulting in protection against *L. major* infection, demonstrating that saliva may provide a protective effect and conferring characteristics for the development of a vaccine against *Leishmania* (90, 99). In a clinical study, was observed that individuals from an endemic area exposed to *P. duboscqi* bite presented high serum levels of INF-γ and decrease of IL-13, IL-5, directing a Th1 profile. Nonetheless, when PBMC of those individuals were exposed to *P. duboscqi* saliva, the most presented a mixed Th1/Th2 response, without a specific polarized profile (82).

In addition to complexes containing salivary components, the biological activity of pharmacological compounds isolated from vector saliva was examined. Adenosine and adenosine monophosphatase are active compounds found in *P. papatasi* saliva, and these factors inhibit the function of dendritic cells via a PGE_2_/IL-10-dependent mechanism to promote a tolerogenic profile that is characterized by the induction of regulatory T cells, which is also related to M2 polarization (73).

Maxadilan (Max) is a vasodilator peptide isolated from the saliva of arthropod vectors, and it reduces CD80 expression, which is responsible for T cell activation, and CCR7, which is involved in the development of adaptive immunity. Max increases CD86 expression on a subpopulation of dendritic cells, which leads to a preferential Th2 type response. Max also promotes an increased production of IL-6, IL-10, and TGF-β, and reduction of the Th1 cytokines, IL-1β, IL-12p70, TNF-α, and IFN-γ (87, 93). Max treatment of *L. major*-infected peritoneal exudate cells increased parasite load because of Th2 polarization and decreased NO production (94). These results suggest that Max acts on M2 polarization, as demonstrated previously for total saliva.

Parasites also play an important role in vector saliva modulation because these pathogens secrete promastigote secretory gel (PSG) in the insect gut. The vector regurgitates PSG with the other salivary components at the moment of blood-feeding (77). *Leishmania mexicana*-PSG regurgitated by *L. longipalpis* exacerbates skin infection via an increase in the recruitment of macrophages to the site of infection (77). PSG also increases parasite load *in vitro* and *in vivo* via increasing arginase activity (77).

In more detail, Giraud et al. (78) demonstrated that PSG exacerbates the inflammatory phase of the early wound response (high levels of cytokines IL-1β, IL-6, IL-10, TNFα and chemokines CCL2, CCL3, CCL4, CXCL2), to induce insulin-like growth factor 1 (IGF1)-signaling and later IGF1-dependent expression of arg-1 in macrophages. As a result, the M2 macrophages promote the effective infection of the parasites in a PGS-dependent manner (78).

**Figure 1:**
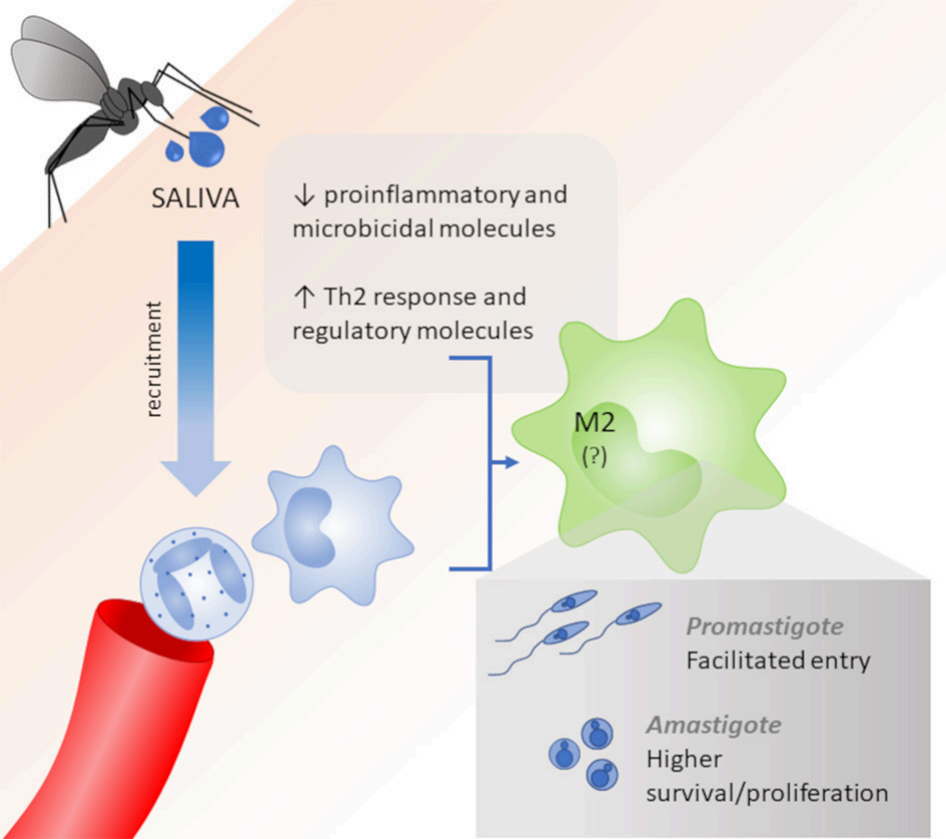
Role of saliva vectors on macrophage polarization. Vector saliva induces the recruitment of neutrophils and macrophages and acts as an immunomodulator to reduce pro-inflammatory and microbicidal molecules and improve Th2 cytokines and regulatory molecules, which lead to a M2 polarization. M2 macrophages allow for the facilitated entry of *Leishmania* promastigotes and higher survival/proliferation of intracellular amastigotes.

In this way, it is noteworthy that some works show that high levels of pro-inflammatory cytokines are induced by saliva components, leading to a Th1 profile, that can act in a host protective way. However, the most of studies inferred that the different salivary components are allied to the immunomodulatory capacity of the parasite, and these components are essential tools in the success of the infection, via the down-regulation of a pro-inflammatory response and reduction of macrophages M1, which are fundamental to parasite elimination and disease resolution. The saliva also up-regulates Th2-standard cytokines and regulatory molecules, which act in M2 polarization, facilitate the promastigote infection and increase the survival and proliferation of intracellular amastigotes (Figure 1).

## M1 and M2 macrophages in *leishmania* infection

The role of macrophage subsets in *Leishmania* infection was not investigated thoroughly. However, the fundamental role of these cells in the development of the lesion support an improved understanding of the M1 and M2 profiles as an important tool in the pathogenesis of leishmaniasis. *In vitro* and *in vivo* studies of the host response to *Leishmania* (Table 3) and therapeutic strategies for modulating key molecules that control cellular activity were performed. We discuss the studies of species that cause the visceral and cutaneous forms of the disease.

**Table 3:** M1/M2 macrophages in leishmaniasis.

**Model**		**Disease**	***Leishmania* specie**	**References**
Mouse	*in vivo*	–	*L. major*	(100)
Mouse	*in vitro*	–	*L. amazonensis*	(101)
Human	NI/*in vitro*	VL	–	(102)
Mouse	*in vitro*	VL	–	(103)
Raw	*in vitro*	–	*L. amazonensis*	(104)
Dog	NI	VL	–	(105)
Dog	NI	VL	*L. infantum*	(106)
Mathematical	–	–	–	(107)
Mouse	*in vitro*	–	*L. major*	(108)
Human	NI	PKDL	–	(109)
Mouse	*in vitro/in vivo*	CL	*L. major*	(110)
Mouse	*in vitro*	–	*L. donovani*	(111)
Mouse	*in vitro*	–	*L. major*	(112)
Mouse	*in vitro*	–	*L. mexicana*	(113)
Mouse	*in vitro*	–	*L. major/L. amazonensis*	(114)

*CL, cutaneous leishmaniasis; NI, natural infection; PKDL, post-kala-azar dermal leishmaniasis; VL, visceral leishmaniasis*.

### M1 and M2 macrophages in cutaneous leishmaniasis (CL)

CL is the most common form of leishmaniasis, with an estimated 600,000 to 1 million new cases worldwide annually. CL causes skin lesions that leave life-long scars and serious disability, and it has become a serious public health problem (3). The primary etiological agents include the *Leishmania tropica* and *L. major* species, in the Old World, and the *L. mexicana* species complex (e.g., *L. amazonensis*), and the subgenus *Viannia*, as the *L. (V.) braziliensis* species complex, in the New World (115).

Patients with CL have higher plasma levels of arg-1, TGF-β and PGE_2_ (116), as wells as, increased arginine in lesions (117), suggesting that M2 macrophages might play a role in the pathogenesis of the disease. In this sense, an *in vitro* study demonstrated that only M2 macrophages allow for *L. major* and *L. amazonensis* growth (114). These authors demonstrated that lipophosphoglycan (LPG) and glycoprotein GP63 of the parasites acted on M2 macrophages and suppressed non-coding RNAs, which left these cells permissive to infection (114). Lee et al. (118) also demonstrated that a non-healing strain of *L. major* efficiently interacted with M2 macrophages (CD206^hi^), which phagocytized the parasite *in vitro* and *in vivo*. However, a strain that produced self-healing lesions was less phagocytosed by M2 macrophages. The authors stated that the preferential infection of M2 cells played a crucial role in the severity of the cutaneous disease.

The role of peroxisome proliferator-activated receptors (PPARs) was investigated in infected macrophages from mouse strains resistant and susceptible to *Leishmania* infection. PPARs are ligand-activated transcription factors that are expressed in macrophages, and regulate the expression of certain genes related to the inflammatory response (119). Odegaard et al. (120) demonstrated that PPARγ-knockout mice have delayed in disease progress, with less footpad swelling and reduced parasitic burden. Importantly, genes preferentially expressed in alternatively activated macrophages, such as *arg-1, Mrc1*, and *Clec7a*, were also decreased in the tissue of PPARγ-knockout mice. These data strongly suggest that PPARγ is required for acquisition and maintenance of the M2 macrophages in *L. major* model (120). Besides that, Gallardo-Soler et al. (121) showed that PPARγ and -δ ligands promote intracellular *L. major* amastigote growth in infected macrophages, and this effect is dependent on both PPAR expression and arg-1 activity, namely suggesting that PPAR ligands promote amastigote growth in M2 macrophages in an arginase-dependent manner (121).

On the other hand, PPAR expression induced the activation of the murine macrophage cell line J774A.1 via polarization toward an M1 profile, with high production of pro-inflammatory mediators (TNF-α, IL-1β, IL-6, TLR4, and ROS), and an increase in microbicide activity against *L. mexicana* (113).

The transmembrane activator and calcium modulator and cyclophilin ligand interactor (TACI) is a key molecule for plasma cell maintenance. This receptor is in the TNF family, and it is required in infections where protection depends on antibody response. Analysis of macrophage phenotype revealed that macrophages adapted the M2 phenotype in the absence of TACI. The levels of M2 markers, IL-4Rα and CD206, were significantly higher in TACI-knockout macrophages than wild-type cells. TACI-knockout mice were unable to control *L. major* infection *in vitro*, which confirms their M2 phenotype, and intradermal inoculation of *Leishmania* resulted in a more severe manifestation of the disease than in the resistant C57BL/6 strain. The transfer of WT macrophages to TACI-knockout mice significantly reduced the disease severity (110).

One fundamental role of TNF was the induction of M1 differentiation and blockade of M2 polarization in the livers of *L. major*-infected mice (100). These authors also described the role of IL-6, which did not interfere with the macrophage phenotype alone, but it was highly expressed in M2 macrophages. The authors suggested that a balance between TNF and IL-6 mediated macrophage polarization in L*. major* infection (100).

Vellozo et al. (122) also demonstrated that the resistance of C57BL/6 mice to *Leishmania* infection was because of their ability to mature macrophages in the peritoneum from M0 to M1. Susceptible mice (BALB/c) exhibit immature peritoneal macrophages and succumb to infection. However, both mouse strains are resistant to *L. braziliensis* infection because in both strains convert M0 to M1 macrophages in this model, despite the incomplete M1 maturation and lower iNOS expression and NO production in BALB/c mice.

The co-culture of mesenchymal stem cells and *L. major*-infected macrophages also induced an event suggestive of M1 polarization, with the induction of inflammatory cytokines and reduction of IL-10 levels (108). This strategy provides new hope for stem cell therapy in the control of *L. major* (108).

SLPI (secretory leukocyte protease inhibitor) is a potent serine protease inhibitor that exhibits antimicrobial and anti-inflammatory functions. The role of SLPI in *L. major* infected-macrophage polarization was investigated (112). SPLI-knockout macrophages produced high levels of iNOS and IFN-γ but failed to contain cutaneous *L. major* infection. This study highlights that a very strong M1 response is detrimental in *L. major* models and causes tissue injury because of exacerbated inflammation. This study suggests that a balance of M1 and M2 macrophage responses influences the outcome of innate host defense against intracellular parasites, and SLPI is critical for the coordination of this balance and the resistance to chronic leishmaniasis (112).

One study of PKDL demonstrated that monocytes from patients exhibited decreased expression of TLR-2/4 and an attenuated generation of reactive oxidative/nitrosative species (109). Patients also exhibited an increased expression of classical M2 markers (arg-1, PPARγ and CD206) in monocytes and lesional macrophages, which indicated the M2 polarization of macrophages. These subsets appeared to sustain disease chronicity, which is a hallmark of Indian PKDL (109).

Two studies showed the development of new drugs addressed the M1/M2 plasticity (101, 104). The first study demonstrated that high antimony dilutions, a homeopathic medicine, potentiated the *L. amazonensis*-induced reduction of pro-inflammatory cytokine (IL-6, IL-12, and IFN-γ) and chemokine (CCL-2 and CCL-4) production in RAW cells. The treatment also induced an increase of the parasites internalization, but there was a reduction of the acid vacuoles, which implied in less elimination of the amastigote forms. The authors suggest that this phenomenon is related to the M2 polarization and regulation of the chronic inflammation events (104).

The second study used crotoxin, which is the main component of *Crotalus durissus terrificus* venom, to treat peritoneal BALB/c mouse macrophages infected with *L. amazonensis* (101). The host cells exhibited an increase in nitric oxide, IL-6 and TNF-α production, that converged into an M1 activation profile, as suggested by their morphological changes (larger spreading), inducing leishmanicidal activity against intracellular parasites, which may be associated with a better prognosis for CL (101).

Siewe et al. (107) proposed a mathematical model that states that *Leishmania* parasite takes advantage of the immune system and invades M1 and M2 macrophages. Simulations of the model demonstrated that the number of M2 macrophages constantly increased and M1 macrophages constantly decreased over the course of the infection, but the sum of the number of M1 and M2 cells reached a steady state, which was approximately the same as the healthy state of the host. The ratio of *Leishmania* parasites to macrophages depends homogeneously on their ratio at the time of the initial infection (107).

### M1 and M2 macrophages in visceral leishmaniasis (VL)

Visceral leishmaniasis is the most aggressive form of the disease, with high rates of mortality (3). Visceral leishmaniasis is generally caused by *L. donovani* and *L. infantum chagasi* species and affects internal organs, such as spleen, liver, and bone marrow (115, 123). It has been shown that blood arginase levels in VL patients are considerably increased (124), while NO levels are decreased (125). In this sense, a recent study demonstrated that monocytes/macrophages of VL patients present reduced oxidative burst and antigen presentation, with a M2 regulatory phenotype, characterized by high CD163, IL-10 and CXCL14 levels (126).

In the same direction, Silva et al. (102) demonstrated that the CD163s molecule was associated with the M2 macrophage phenotype, and it may be used as a biomarker of the clinical parameters of human VL severity. *L. donovani* also promotes the output of monocytes with a regulatory phenotype from the bone marrow that function as safe targets for the parasite (127). These data are consistent with analyses of dogs with VL, that demonstrated a higher number of M2-polarized macrophages compared to healthy dogs (106). The authors concluded that the predominance of the M2 phenotype (high CD163 labeling) in VL dogs favored the multiplication of *L. infantum* in the skin, spleen and lymph nodes (106).

Similarly, Chan et al. (128) suggest that the regulation of the macrophage phenotype is induced by the parasite, since PPAR expression is induced by parasitic infection. The authors showed that *L. donovani* activation of PPARγ promotes survival, whereas blockade of PPARγ facilitates removal of the parasite. Thus, *Leishmania* parasites harness PPARγ to sustain the M2 phenotype and increase infectivity of visceral disease (128).

A recent study has also shown the role of mammalian target of rapamycin (mTOR) in M2 macrophage polarization for *Leishmania* survival (129). The authors observed that *L. donovani-*infection activated host mTOR pathway, resulting in reduced expression of M1 macrophage markers (ROS, NO, iNOS, NOX-1, IL-12, IL-1β, and TNF-α), and increased expression of M2 macrophage markers (arg-1, IL-10, TGF-β, CD206, and CD163), favoring the *Leishmania* survival inside macrophages. Thus, they concluded that mTOR plays a crucial role in M2 macrophage polarization and parasite persistence in *L. donovani* infection (129).

These results correlated with studies performed in *L. donovani*-infected Syrian hamsters, in which the spleen environment was inflammatory with a high production of types I and II IFN. However, IFN-γ did not direct M1 macrophage polarization in this model, which allowed for parasite growth and the expression of counter-regulatory molecules, such as arg-1 (103).

A different group demonstrated that macrophages cultured from the lymph nodes of VL dogs exhibited low arg-1 activity and high NO and PGE_2_ production compared to uninfected dogs (105). These authors suggested that M1 macrophages were participating in the immune response in the lymph nodes of these animals (105). Attenuated *L. donovani* induced innate immunity via the classical activation of macrophages (M1), with upregulation of IL-1β, TNF-α, IL-12 and downregulation of IL-10, YM1, Arg-1, and MRC-1 genes, which led to the generation of protective Th1 responses in BALB/c mice (111).

This controversy may be explained by the fact that different patterns of gene expression are found in macrophages at different time points (130). A recent study demonstrated that the early response against *L. donovani* infection was distinguished by the increase in of Th1 markers and M1-macrophage activation molecules (IFN-γ, Stat1, Cxcl9, Cxcl10, Ccr5, Cxcr3, Xcl1, and Ccl3). However, this activation was not protective because the parasitic burden increased over time. There was no marked overlap of macrophage phenotypes at intermediate times of infection, and the overexpression of these Th1/M1 markers was restored later in the chronic phase without parasitic burden control (130).

**Figure 2:**
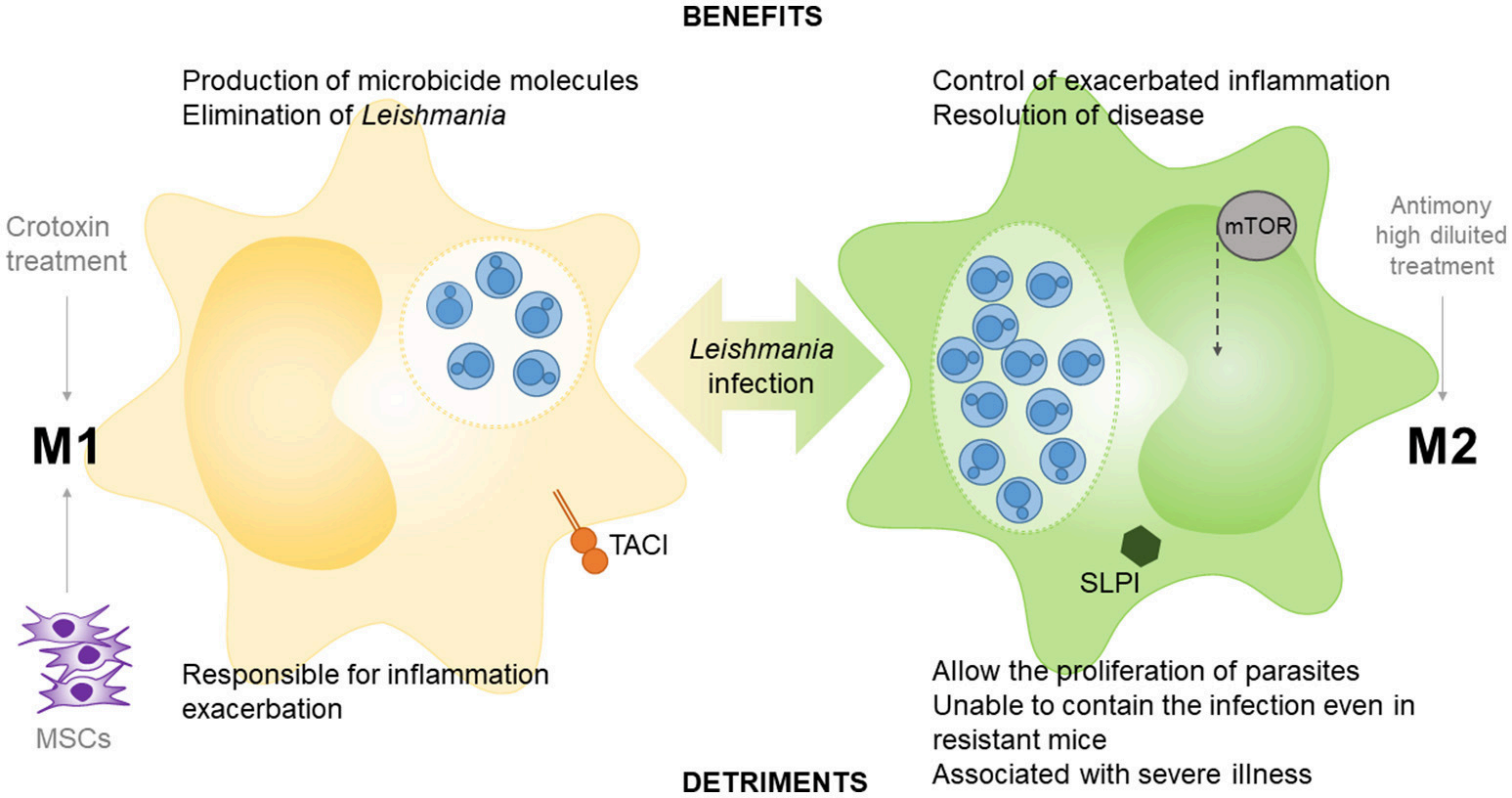
Role of M1 and M2 macrophages in *Leishmania* infection. TACI (transmembrane activator and calcium modulator and cyclophilin ligand interactor), peroxisome proliferator-activated receptors (PPARs) and mesenchymal stem cells (MSCs) participate in M1 polarization in macrophage-leishmania studies, as well as the crotoxin treatment. The epithelial and myeloid-derived serine protease inhibitor (SLPI), mammalian target of rapamycin (m-TOR) and the treatment with high diluted antimony participate in M2 polarization in leishmania models.

Together, these results demonstrate that there are no “good guys” and “bad guys” in the polarization of macrophages following infection by *Leishmania* parasites. Therefore, a balance between the potent microbicidal response of M1 macrophages and the potential regulation by M2 macrophages may be key to the success of overcoming leishmaniasis (Figure 2).

## Conclusion

The collected works suggest that vector saliva plays an immunomodulatory effect on macrophages, which leads to the polarization of macrophages to an M2 phenotype. Therefore, vector saliva may contribute to an increase in the infectivity and persistence of the parasite during the initial periods of infection. Different *Leishmania* species exhibit virulence factors that can subvert the microbicidal mechanisms of the host to favor its proliferation because of the M2 polarization. However, M1 macrophages act during later periods of the infection and trigger an exacerbated immune response that leads to a worsening of the lesions, despite the role of these cells as powerful microbicidal agents. Therefore, a balance between the initial microbicidal response (M1) followed by a restorative response (M2) at later periods of time would provide the utmost benefit for the host. Further studies are necessary to fully elucidate the balance between these two main macrophages populations in the control of *Leishmania* infection.

## Author contributions

FT-P study design, literature review, data collection and manuscript writing. BB, JA, MG, AC, and MM-S literature review, data collection and manuscript writing. IC-C and JB text correction and organization. WP study design, text correction and organization.

### Conflict of interest statement

The authors declare that the research was conducted in the absence of any commercial or financial relationships that could be construed as a potential conflict of interest.

## Abbreviations

Arg: arginase
CL: cutaneous leishmaniasis
IL: interleukin
IFNγ: interferon gamma
IGF1: insulin-like growth factor 1
iNOS: inducible nitric oxide synthase
LPS: lipopolysaccharide
Max: maxadilan
MCL: mucocutaneous leishmaniasis
mTOR: mammalian target of rapamycin
NO: nitric oxide
PKDL: post-kala-azar dermal leishmaniasis
PPAR: proliferator-activated receptors
PSG: promastigote secretory gel
ROS: reactive oxygen species
SGE: salivary gland extract
SGH: salivary gland homogenate
SGL: salivary gland lysate
TACI: transmembrane activator and calcium modulator and cyclophilin ligand interactor
TGFβ: transforming growth factor beta
Th: T helper
TLR: toll like receptor
TNFα: tumor necrosis factor alpha
Treg: T regulatory
and VL: visceral leishmaniasis.
